# Exploring the Role of DeepSeek in Addressing Common Patient Concerns About Colorectal Cancer: A Commentary

**DOI:** 10.7759/cureus.99470

**Published:** 2025-12-17

**Authors:** Sridhar V.N.S. Kocharlakota

**Affiliations:** 1 Internal Medicine, Oncology, Gastroenterology, Cardiology, Government Medical College, Srikakulam, Srikakulam, IND

**Keywords:** artificial intelligence, asia-pacific region, colorectal cancer, deepseek, early detection

## Abstract

Since its inception, Artificial Intelligence (AI) has demonstrated its utility across diverse scientific and clinical domains. One such AI chatbot, developed by a Chinese AI company and widely used across the Asia-Pacific region, is DeepSeek AI. As it is well known, colorectal cancer is a common malignancy of the gastrointestinal tract that usually produces symptoms (similar to many benign gastrointestinal disorders) such as a change in bowel habits, including persistent diarrhea and/or constipation; occult blood in stool causing gradual iron deficiency anemia and pallor; bloating; generalized abdominal discomfort; and fatigue, most of which are usually overlooked or result in a missed diagnosis of the condition unless there is a high level of suspicion by the clinician. Many individuals, especially in the Asia-Pacific region, who experience such symptoms and/or have a positive family history of colorectal cancer, are becoming increasingly skeptical and are turning to AI chatbots such as DeepSeek AI to explore their health concerns rather than consulting a healthcare professional. This article explores and records such common patient concerns and the responses generated by DeepSeek AI, starting from recognizing the early symptoms and signs, causes of colorectal cancer, strategies for prevention and screening, diagnostic modalities, treatment options, and reducing recurrence risk. The responses generated by DeepSeek AI were documented along with the patient’s concerns to facilitate further assessment of their clinical relevance. Finally, a broad review and validation was performed on the responses given by DeepSeek AI, including the authenticity of the response and its relevance to the current health care guidelines. The article ends with a small discussion of the future scope and improvements in generating responses that align with current healthcare guidelines and clinical standards, ensuring safer and more context-aware integration of DeepSeek AI in addressing patient concerns.

## Editorial

DeepSeek AI is an Artificial Intelligence (AI) chatbot developed by the Chinese AI company DeepSeek, which develops LLMs. Since its launch in January 2025, the DeepSeek-R1 model has surpassed other existing AI chatbots, such as ChatGPT, as the most downloaded AI app and is widely in use in the Asia-Pacific region. Machine learning techniques such as the Mixture of Experts and reinforcement learning, etc., have helped DeepSeek-R1 perform on par with GPT-4o and OpenAI o1a [1].

Colorectal cancer is the third most common cancer in both men and women, and its incidence has been increasing owing to various factors such as lifestyle changes and dietary habits, or common risk factors such as obesity, smoking, and alcohol consumption [2].

Colorectal cancer is one of the most common malignancies of the gastrointestinal tract and produces early signs and symptoms (similar to a variety of benign gastrointestinal disorders), such as changes in bowel habits, including persistent diarrhea and/or constipation; occult blood in stool, causing gradual iron deficiency anemia and pallor; bloating; generalized abdominal discomfort; and fatigue. Most of these symptoms are usually overlooked by the patient or could even result in a missed diagnosis of the condition unless there is a high index of suspicion by the clinician, contributing to anxiety and overthinking among individuals with such symptoms. In addition, patients with a known family history of colorectal cancer may grow very skeptical, making them more inclined and drawn towards using AI chatbots such as DeepSeek AI to address their concerns, probably due to the anxiety of visiting a healthcare professional [3].

DeepSeek AI was used to explore and analyze what patients commonly asked or worried about, and their responses were recorded (Table 1).

**Table 1:** Questions Asked and Responses Given by DeepSeek AI About Colorectal Cancer Summary of common patient questions and corresponding responses generated by DeepSeek AI regarding colorectal cancer awareness, diagnosis, and management. AI: Artificial Intelligence; MSI-H: Microsatellite Instability-High; dMMR: Deficient Mismatch Repair

Common Patient Questions	Responses Generated by DeepSeek AI
1. What are the early signs and symptoms of colorectal cancer?	Colorectal cancer often develops silently in early stages, but some symptoms may appear as the disease progresses. Early detection is crucial — here are key warning signs: Changes in bowel habits such as persistent diarrhea or constipation lasting more than two weeks, narrower stools, or a feeling of incomplete emptying. Blood in stool (even if thought to be hemorrhoids). Unexplained abdominal discomfort, weight loss, fatigue, or iron-deficiency anemia. People with a family history plus symptoms may need earlier screening.
2. What are the risk factors and causes of colorectal cancer?	Colorectal cancer develops due to a combination of genetic, lifestyle, and environmental factors. Most cases arise from precancerous polyps in the colon or rectum. Non-modifiable risk factors include age over 50 years, family history, hereditary syndromes, personal history of polyps, or inflammatory bowel disease. Modifiable risk factors include high intake of red or processed meat, low fiber intake, excessive alcohol, smoking, and physical inactivity.
3. What are the ways to prevent colorectal cancer and reduce risk?	Colorectal cancer is largely preventable through lifestyle modification and screening. Regular screening via colonoscopy every 10 years, fecal immunological testing, or CT colonography. Diet rich in fiber, whole grains, fruits, and omega-3 fatty acids. Increased physical activity, smoking and alcohol cessation, and management of chronic conditions. Genetic testing and early screening if a first-degree relative had colorectal cancer.
4. What are the stages of colorectal cancer and how are they determined?	Colorectal cancer is staged from 0 to IV based on tumor spread. Stage 0: Cancer cells confined to inner lining. Stage 1: Tumor invades deeper layers but not beyond the wall. Stage 2: Cancer through colon wall but no lymph node involvement. Stage 3: Spread to nearby lymph nodes. Stage 4: Metastasis to distant organs (liver, lungs, abdomen). Diagnosis uses colonoscopy with biopsy, CT/MRI/PET scans, chest X-ray, and CEA marker testing.
5. What treatment modalities are available?	Management depends on stage and site. Surgery: Removal of tumor and nearby lymph nodes. Chemotherapy: Pre- or post-surgical or for advanced disease. Radiation therapy: For rectal cancers, pre-surgery or palliative use. Immunotherapy: For MSI-H/dMMR advanced cancers. Treatment is personalized by oncologist based on staging and genetic profile.
6. Can colorectal cancer metastasize if left untreated? Can it be stopped?	Yes, colorectal cancer can metastasize if untreated. Early detection and removal of precancerous polyps prevent progression. Even stage IV cancer may be controlled with chemotherapy, targeted therapy, or immunotherapy.
7. Can colorectal cancer recur after treatment? How can recurrence risk be reduced?	Recurrence may occur locally or in distant organs (liver, lungs, peritoneum). Most occur within two to three years of treatment. Risk reduction includes complete resection, adjuvant chemotherapy if indicated, regular follow-up (blood tests, imaging, colonoscopy), and maintaining a healthy lifestyle (exercise, fiber-rich diet, no smoking or alcohol excess).

Large language models, such as DeepSeek, are becoming increasingly important in patient education; however, in their current form, they are not yet reliable enough to serve as primary sources of medical guidance. They hold great potential for simplifying complex medical information and improving patient engagement and participation in awareness of colorectal cancer. However, this potential must be balanced against the risks of misinformation, inconsistent response generation and accuracy, and lack of clinical oversight.

The above responses from DeepSeek AI were concise, sympathetic, and well organized, helping patients easily understand complicated information. Its constant emphasis on the importance of speaking with an oncologist highlights the value of early intervention and professional evaluation. These characteristics demonstrate how conversational AI can promote prevention-focused communication and increase cancer awareness among the target audience. For the most part, DeepSeek AI-generated responses aligned well with current healthcare guidelines, showing consistency with existing screening recommendations and symptom recognition for colorectal cancer. However, it is important to address and anticipate a few key limitations before broader clinical adoption [3].

One of the most concerning issues with DeepSeek and similar large language models is their tendency to generate reasonably good-looking yet factually incorrect or suboptimal medical information, a phenomenon commonly referred to as hallucination. Hazra et al. evaluated four state-of-the-art models for their diagnostic correctness and quality of explanation by assessing four metrics: hallucination rate, reasoning score, anatomical correctness, and grounding deviation score. It has been shown that, even though few models have achieved moderate diagnostic accuracy, there are concerns regarding hallucination, incorrect reasoning, and shallow patterns. This becomes troublesome in the context of patient education, where individuals may accept confident-sounding but unvalidated responses as authoritative medical guidance [4].

Although patients are consistently advised to speak with a healthcare provider, not all patients heed this guidance, particularly those experiencing anxiety about medical visits or living in underserved regions where access to healthcare poses significant challenges. Although these responses are backed by good intentions, they may inadvertently reassure patients with concerning symptoms, potentially postponing timely diagnostic evaluation when early detection could make a difference in the prognosis. In addition, patients with a positive family history and/or concerning symptoms can increasingly turn to AI chatbots for initial guidance before consulting a healthcare professional, which can increase the risk of delayed detection and/or disease progression.

In addition, another fundamental limitation consistent with previous studies on healthcare chatbots is the absence of authentic peer-reviewed references to support DeepSeek's recommendations. Without citing specific studies, guidelines, or evidence levels, it is difficult for patients or clinicians to verify responses or assess the strength of the supporting evidence. In addition, response variability adds to these concerns; subtle changes in how a question is phrased and/or the input prompt can produce materially different answers and responses, thus questioning the reliability and consistency of the information delivered [3].

Recent reports have mentioned that DeepSeek surpassed ChatGPT and Google Gemini by ten times since late January 2025 in terms of global search queries [5]. Zeng et al., in their recent analysis, have mentioned that over 300 Chinese hospitals have deployed DeepSeek into their clinical tasks, diagnostic services, decision-making, and hospital management systems, and have raised concerns over inadequate evaluation, hallucination, and patient safety [5].

These patterns have practical implications for key stakeholders. For clinicians, AI-generated content should be positioned as an adjunct, something that may prepare patients for consultations but cannot replace them. Developers must prioritize transparency, guideline alignment, and the incorporation of uncertainty signals to ensure that users understand when an answer may be incomplete or unreliable. Policymakers and regulatory bodies should consider validation frameworks, especially before models such as DeepSeek are widely deployed in hospital systems worldwide. Without such safeguards, there is a risk that conversational AI could unintentionally normalize inaccurate or oversimplified cancer-related guidance for patients.

A reasonable counterargument is that conversational AI might significantly increase access to basic cancer information, particularly in areas where specialist availability is limited or where stigma and fear of visiting a healthcare professional may delay medical consultation. For some individuals, interacting with an AI system may be less intimidating than speaking with a clinician. This perspective reflects a legitimate benefit that should not be overlooked. However, the benefits of interacting with conversational AI systems should not compromise the responsibility to ensure accuracy, nor should they justify replacing evidence-based medical communication.

A more balanced approach would be to improve factual accuracy and confidence, transparently communicate with users about the limitations and lack of stating responses that are well-supported by evidence, and incorporate rigorous clinical validation, peer-reviewed research, and oversight. Tools such as DeepSeek should be considered supplementary rather than primary sources of medical information until they meet the established clinical, ethical, and evidence-based standards. While AI may eventually contribute meaningfully to how patients learn about colorectal cancer, its most responsible role at present is to support, not replace, qualified medical professionals.

In summary, while DeepSeek is a promising tool for improving patient understanding of colorectal cancer, its current limitations in terms of accuracy, personalization, and transparency make it unsuitable for replacing clinically validated professional medical consultations. The role of such conversational AI tools, at this stage, is to support, not substitute, clinicians by helping patients engage with information more confidently and arrive better prepared for health care consultations. As development continues, rigorous clinical validation, peer-reviewed research, ethical oversight, and clear communication regarding uncertainty and evidence sources are required. Until the required standards are met, DeepSeek and similar systems should be used cautiously and under guidance, ensuring patient safety as the utmost priority.
